# Medical Management of Aortic Disease: If They Don’t Need Surgery, What Do They Need?

**DOI:** 10.14797/mdcvj.1192

**Published:** 2023-03-07

**Authors:** Mujtaba Saeed, Maan Malahfji

**Affiliations:** 1 Houston Methodist DeBakey Heart & Vascular Center, Houston, Texas, US

**Keywords:** aortitis, aortic aneurysm, aortic dissection

## Abstract

Management of aortic disease has evolved significantly over the past few decades. A preemptive diagnostic approach with a multidisciplinary team and shared decision-making has led to improved clinical outcomes. Surgery is the cornerstone of management for most aortic conditions; however, medical therapy is now an important adjunctive therapy in most if not all patients. Herein, we review the role and evidence behind medical management of patients with aortic disease.

## Introduction

Aortic diseases encompass a heterogenous spectrum of clinical presentations. Many pathologies are detected incidentally, while some develop insidiously and present acutely. Hyperacute and acute presentations can be deadly and include aortic dissections, rupture of an aneurysm, intramural hematomas, and penetrating aortic ulcers. The spectrum of aortic disease also includes connective tissue disorders and genetic syndromes as well as inflammatory aortic diseases. Management of aortic diseases historically has hinged on surgical intervention. However, medical management has evolved over the years and now plays an important role in the management of most aortic diseases. In patients who undergo surgery, medical therapy also has become key in pre- and postoperative care. Patient characteristics, comorbidities, underlying pathology, and the anatomical area of involvement are all factors that influence the need and type of recommended medical management. The American College of Cardiology/American Heart Association (ACC/AHA) recently released aortic disease guidelines that highlight the importance of medical therapy in most aortic conditions.^1^ In this article, we review the current evidence for medical therapies of various aortic diseases.

## Aortic Aneurysms

Medical management of patients with thoracic aortic aneurysms (TAA) and abdominal aortic aneurysms (AAA) includes controlling cardiovascular risk factors, aggressive blood pressure control, patient education, lifestyle modification, genetic counseling, and serial imaging to optimize treatment, timing of surgery, and detection of other sites of aortic aneurysm development. A primary objective of medical management is to reduce the growth rate of aneurysms and the risk of complications.

In patients with sporadic and degenerative aortic aneurysms, clinical trials of medical therapy are scarce because most studies have focused on genetic aortic conditions. In patients with TAA who have an average systolic blood pressure ≥ 130 mm Hg or diastolic blood pressure ≥ 80 mm Hg, use of antihypertensive agents is recommended to reduce the risk of cardiovascular events.^1^ Beta-blockers are recommended since they decrease heart rate, blood pressure, and the force of ejected blood on the aortic wall.^2^ In addition, angiotensin receptor blockers (ARBs) are reasonable adjuncts to beta blockers in achieving target blood pressure goals. ARBs are postulated to decrease aneurysm expansion by inhibiting intracellular mediators in the transforming growth factor-β signaling cascade and reducing matrix metalloproteinase (MMP) levels.^3^ MMPs have increased expression in aneurysms and promote proteolysis, hence propagating aneurysmal expansion and increasing the risk of dissection or rupture.^4^ However, there is no randomized data that shows a reduction in sporadic TAA size or growth rate with these medications.

There is data on the protective effects of statins in TAAs, and the mechanism may be credited to anti-inflammatory properties and inhibition of MMPs.^5^ Statin use is also associated with a decrease in the incidence of dissection, rupture, surgical intervention, and death.^5,6^ In a retrospective study of patients who underwent thoracic endovascular repair for aneurysmal disease, patients who were treated with statins preoperatively had a significantly lower rate of perioperative complications and 5-year mortality.^7^

Cigarette smoking is a strong risk factor contributing to aneurysm formation, growth, and rupture. Cessation of smoking and avoidance of secondhand smoke should be encouraged in all patients. A retrospective study of three million patients demonstrated smoking to be a major risk factor for AAA, with a positive correlation to the quantity and duration of smoking and an inverse relation with years after smoking cessation.^8^ Blood pressure control helps reduce the risk of cardiovascular events such as myocardial infarction and stroke in patients with AAA.^1^ Antihypertensive therapies involving β-blockers are commonly employed for AAA, and observational data on statin use have been favorable as well.^9,10^ The current ACC/AHA aortic disease guidelines recommend the use of statins in patients with AAA.^1^ Studies show that statins are associated with a reduction in the risk of AAA rupture as well as mortality in patients with ruptured AAA.^11^ Antiplatelet therapy with low-dose aspirin is also recommended for patients with atherosclerotic TAA and AAA since atherosclerotic aortic diseases are considered a coronary artery disease equivalent.^12^

The use of doxycycline (a nonspecific MMP inhibitor) has shown efficacy in preventing TAA in a mouse model of Marfan syndrome, and its use is hypothesized to be beneficial for patients with Marfan syndrome; however, no human studies have been conducted.^13^ In contrast, fluroquinolones are associated with a potential increased risk of aortic dissection and rupture.^14^ A large population study associated fluroquinolone use with a very small increase in the rate of aortic aneurysm or dissection.^15^

Lifestyle modifications are an important aspect of aortic disease management. Exercise and activity limitation are particularly relevant in patients with aortic aneurysm. There are instances of aortic dissection and rupture in weightlifters with a moderate aortic aneurysm size (4 to 5 cm), suggesting that heavy weightlifting and bursts of strenuous exercise that lead to a rapid and detrimental increase in blood pressure should be avoided.^16^ In addition, there is an association between sudden emotional stress and TAA rupture, presumably due to sudden increases in blood pressure.^17^

## Genetic Connective Tissue Diseases

Genetic diseases that involve the aorta most often involve the thoracic portion, and these patients have a higher risk of dissection and rupture. Because aortic aneurysms arise earlier in these patients, it is important to identify those at risk of familial aortic aneurysmal disease (Table 1).^1^

**Table 1:** Risk factors for familial thoracic aortic aneurysm (TAA).


TAA and syndromic features of Marfan Syndrome, Loeys-Dietz syndrome, or vascular Ehlers-Danlos syndrome

TAA at < 60 years of age

Family history of TAA, intracranial or peripheral aneurysm

History of unexplained sudden death at a young age in a first- or second-degree relative


Marfan syndrome is an autosomal dominant connective tissue disease caused by a mutation in the *FBN1* gene that encodes fibrillin-1.^18^ As fibrillin-1 regulates the activation and signaling of cytokine transforming growth factor β (TGF-β), studies in a mouse model of Marfan syndrome showed that a deficiency of fibrillin-1 was associated with excessive signaling by TGF-β.^19^ Beta-blockers have been recommended in patients with Marfan syndrome to reduce the rate of aortic dilatation.^20^ Angiotensin receptor blockers also have shown efficacy in slowing the rate of aortic root dilation in patients with Marfan syndrome owing to TGF-β antagonism.^3,19^ A randomized controlled trial involving children and young adults with Marfan syndrome who received irbesartan or placebo demonstrated a decrease in the rate of aortic dilation.^21^ Another trial investigated the use of losartan in adult patients and also showed a reduction in aortic dilation rate.^22^ However, in a 2014 randomized controlled trial, no significant difference in the rate of aortic root dilation was found between β-blocker and ARB therapy.^23^ A meta-analysis showed that combination treatment with β-blockers and ARBs led to a lower rate of aortic dilation.^24^

Medical management of Loeys-Dietz syndrome is similar to patients with Marfan syndrome and relies on β-blocker use.^1^ Based on studies of mouse models, the use of ARBs is also considered,^25^ but there are no randomized trials in humans to show a reduction in aortic diameter growth or the risk of aortic events.

Vascular Ehlers-Danlos syndrome (vEDS) is another inherited autosomal dominant connective tissue disease that involves the aorta, leading to progressive aneurysm formation or spontaneous dissection and/or rupture.^26^ Medical therapy is focused on managing and preventing vascular complications. Prophylactic measures include blood pressure control by β-blockers and atherosclerotic risk factor reduction.^27^ There have been trials on the use of celiprolol (a long-acting β-1 adrenoreceptor antagonist and partial β-2 agonist) to prevent dissections and ruptures, with one study showing a three-fold decrease in fatal vEDS-related events and another study demonstrating that a daily dose of celiprolol (400 mg) provided optimal protection.^28,28,29,30^

## Aortic Dissection

Emergent surgery is the standard of care in patients with ascending aortic dissections.^1,31,32^ Initial medical management involves anti-impulse therapy and pain management. Beta-blockers decrease stress on the aortic wall by reducing heart rate and blood pressure, hence slowing the progression of dissection and maintaining end-organ perfusion.^33,34^ It is preferred to reach a target heart rate < 70 bpm and systolic blood pressure between 100 mm Hg and 120 mm Hg for adequate organ perfusion.^35^ Beta-blockers have a class I recommendation to lower blood pressure in patients with aortic dissection (AD). In addition, intravenous nitroprusside, nicardipine, and nitroglycerin can be used as adjuncts.^33,34^ Suzuki et al. examined data of 1,301 patients from the International Registry of Acute Aortic Dissection and showed that type A and type B AD patients who were discharged on β-blockers had better outcomes, while calcium channel blockers were associated with longer survival in type B AD.^36^ In patients with contraindications to β-blockers, esmolol or nondihydropyridine calcium channel blockers can be used, while labetalol also offers an advantage of alpha and beta antagonism.^34^

**Figure 1:**
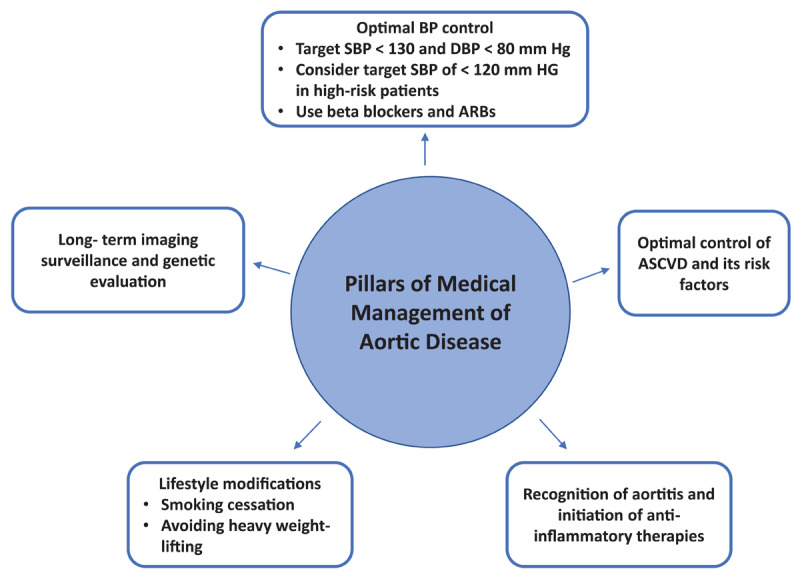
Summary figure of aortic disease management. BP: blood pressure; SBP: systolic blood pressure; DBP: diastolic blood pressure; ARBs: angiotensin receptor blockers; ASCVD: atherosclerotic cardiovascular disease

In addition to lowering blood pressure, it is important to provide adequate pain relief to patients. Uncontrolled pain can activate the sympathetic response, resulting in a high blood pressure, heart rate, and propagation of the tear.^34^ Patients with AD also require long-term medical management. Oral antihypertensive regimens that include β-blockers, ARBs, and angiotensin-converting enzyme inhibitors improve long-term outcomes in patients.^37^ Figure 1 provides an overview of the main targets of medical therapy in patients with aortic disease.

## Inflammatory Aortic Diseases

Aortitis is defined as a nonatherosclerotic and noninfectious inflammatory process that involves the tunica media with or without involving the adventitia.^38^ Evidence is scarce on the incidence of inflammatory aortic diseases, or aortitis, primarily due to their multifactorial nature and lack of a definitive classification.^38,39^ However, we have epidemiological data on the most common causes such as giant cell arteritis (GCA) and Takayasu arteritis (TA).^39^

According to a study of 255 patients who underwent surgery for thoracic ascending aortic aneurysm, the most common histopathological pattern in patients with aortitis (n = 35) was granulomatous giant cell (n = 20).^40^ The study also associated aortitis with advanced age, female gender, and a higher prevalence of cardiovascular risk factors.^40^ Another study that analyzed resected thoracic aorta specimens of 788 patients with aortitis found that GCA was the most common histopathological finding (76.9%) and more frequent in women. However, out of 38 patients with noninfective aortitis, 92.3% had isolated aortitis with no established systemic disease.^41^

Aortitis commonly involves the thoracic aorta, resulting in aneurysmal dilation of the aortic root and/or ascending aorta owing to an inflamed and thin aortic wall.^42^ Although AD and rupture can occur, massive fibrosis can be a protective factor as seen in the healing phase.^31,40,42^ Another rare cause of aortitis is IgG4-related disease, which can cause lymphoplasmacytic thoracic aortitis and chronic periaortitis involving the abdominal aorta; the latter can be associated with retroperitoneal fibrosis.^43^

Surgical repair of an inflamed aortic aneurysm may not have favorable results due to the fragility of aortic tissue.^41^ Hence, immunosuppressive agents are the primary treatment of choice. High-dose oral glucocorticoids (prednisone at 40 - 60 mg/dL) and/or intravenous pulse steroid therapy are started as early induction therapy.^44^ Disease-modifying antirheumatic drugs (DMARD) are used as adjuncts in select patients who are at risk of relapse, develop glucocorticoid-related adverse effects, or require prolonged glucocorticoid therapy.^45,46,47^ Methotrexate and tocilizumab are frequently used DMARDs in combination with steroids for the management of GCA and TA.^48,49^ Nonbiological DMARDs (eg, methotrexate, hydroxychloroquine, azathioprine, sulfamethoxazole, and leflunomide) are first-line agents, and biological DMARDs (eg, tocilizumab or tumor necrosis factor inhibitors) are considered second-line agents in TA.^44^

**Figure 2:**
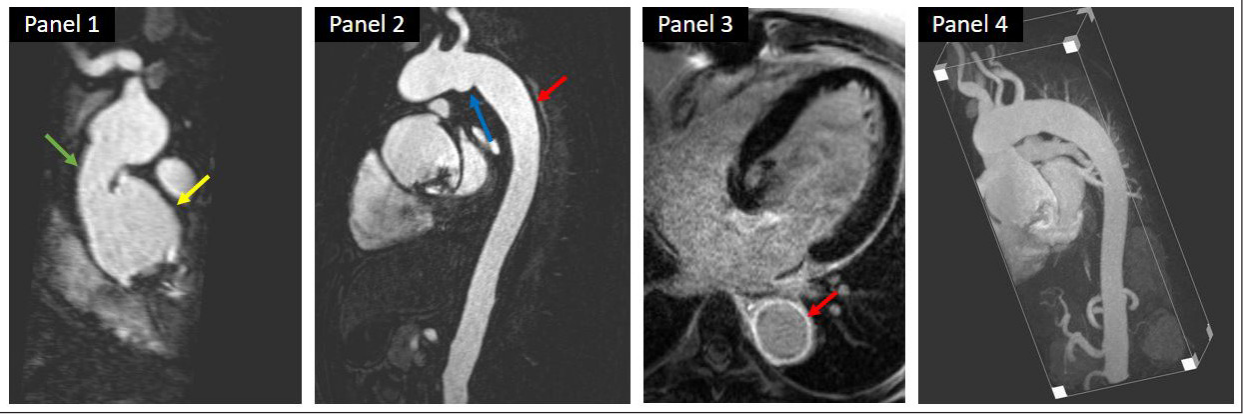
A case highlighting the importance of medical management in a patient with aortic disease. A 47-year-old man with a history of ascending aortic grafting and aortic valve replacement who underwent follow-up magnetic resonance angiography. The study showed a large aneurysmal formation (yellow arrow) just proximal to the ascending aortic graft (green arrow). Another small aneurysm (blue arrow) was seen at the aortic isthmus along with significant aortic wall thickening and enhancement on contrast angiography, consistent with aortitis (red arrows). Review of the histopathology slides from the aortic wall demonstrated wall thickening and extensive fibrosis along with a dense inflammatory infiltrate, with features suggestive of IgG4-related disease. The patient was started on immunomodulators and his follow-up imaging showed stable aortic dimensions.

Initial medical therapy for active TA and GCA reduces the active inflammatory state. Elective surgical repair of TAA for patients with TA and GCA should be delayed until the acute inflammatory state is treated and quiescent (Figure 2).

## Future Directions

There are many gaps in the current literature on medical management of aortic diseases, although a significant number of trials are being conducted that may provide clarity on the optimal approach. Active randomized controlled trials are evaluating the benefits of physical conditioning in patients with aortic diseases, especially postoperative patients.^50^ The incorporation of nonpharmacological/lifestyle modifications in tandem with a focus on improving patient-reported health-related quality of life is of paramount importance and may improve outcomes.^51^ Since data does not provide distinct risk stratification on gender differences and race-based differences in mortality and morbidity, it will be important for future studies to focus on these aspects to improve outcomes across a spectrum of demographics. The mechanical properties of normal and diseased aortic tissue are also of great research interest. Studies using 4-dimensional magnetic resonance imaging to evaluate the wall shear stress and other aspects of aortic flow may provide important data crucial for a tailored management of these patients.^52^ All of the above are pertinent areas that need further research.

## Conclusion

Although surgery is the definitive treatment for the majority of aortic diseases, medical management has evolved significantly to supplement surgery. Control of blood pressure, cholesterol, lifestyle factors, and comorbidities are all important and meaningful targets to optimize outcomes in aortic disease patients. Further research is needed to develop effective medical therapies to prevent and treat both sporadic and genetic aortic aneurysms.

## Key Points

- While surgery remains the mainstay of management of many aortic diseases, optimal medical therapy and control of risk factors can improve patient outcomes.
- Early screening of at-risk patients and initiation of medical therapy can reduce complications in patients with aortic disease.
- Further research is needed to develop and test medical therapy for various aortic diseases.

## CME Credit Opportunity

Houston Methodist is accredited by the Accreditation Council for Continuing Medical Education (ACCME) to provide continuing medical education for physicians.

Houston Methodist designates this Journal-based CME activity for a maximum of *1 AMA PRA Category 1 Credit*™. Physicians should claim only the credit commensurate with the extent of their participation in the activity.

Click to earn CME credit: learn.houstonmethodist.org/MDCVJ-19.2.
